# Kala-azar Epidemiology and Control, Southern Sudan

**DOI:** 10.3201/eid1404.071099

**Published:** 2008-04

**Authors:** Jan H. Kolaczinski, Andrew Hope, Jose Antonio Ruiz, John Rumunu, Michaleen Richer, Jill Seaman

**Affiliations:** * Malaria Consortium, Kampala, Uganda; † London School of Hygiene & Tropical Medicine, London, UK; ‡ Malaria Consortium, Southern Sudan Office, Juba, Sudan; § World Health Organization, Juba, Sudan; ¶ Ministry of Health, Government of Southern Sudan, Juba, Sudan; # International Medical Relief Fund, Watsonville, California, USA

**Keywords:** Leishmaniasis, *Leishmania donovani*, visceral, kala-azar, epidemiology, control, Southern Sudan, dispatch

## Abstract

Southern Sudan is one of the areas in eastern Africa most affected by visceral leishmaniasis (kala-azar), but lack of security and funds has hampered control. Since 2005, the return of stability has opened up new opportunities to expand existing interventions and introduce new ones.

Visceral leishmaniasis (kala-azar) is a deadly disease caused by the *Leishmania* protozoan parasite and transmitted through the bite of sandflies. Without prompt appropriate treatment, as many as 95% of kala-azar patients die, resulting in at least 50,000 deaths per year worldwide (*1*). Each death equates to a loss of 34 disability-adjusted life years (*2*). Continuous and large-scale control of kala-azar in the 2 foci of Southern Sudan has been hampered by war and instability. However, after the Comprehensive Peace Agreement between North and Southern Sudan was signed on January 9, 2005, the return of relative stability to Southern Sudan is now opening up new opportunities for supporting and improving healthcare delivery. To raise international awareness of kala-azar in Southern Sudan, we reviewed the available data and interventions and report the current status and plans for control of kala-azar.

## The Review

**Figure 1:**
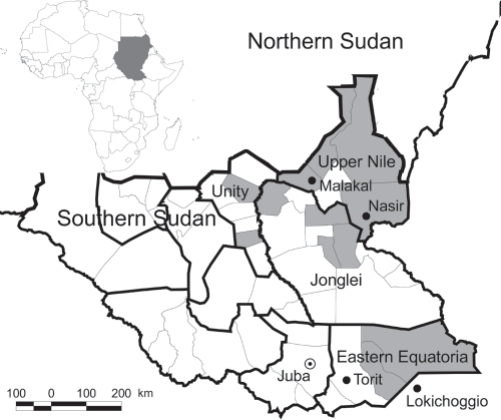
Map of Southern Sudan showing the 2 foci of visceral leishmaniasis. Shaded areas represent those counties where primary cases were reported from January through June 2007. Inset shows location of Sudan in Africa. (Adapted from World Health Organization, Southern Sudan Health Update, July–August 2007.)

Kala-azar occurs in 2 foci (Figure 1) and is caused by *L. donovani.* In the northern focus (Upper Nile, Jonglei, and Unity states), *Phlebotomus orientalis* is the vector; in the southern focus (parts of Eastern Equatoria state), *P. martini* is the vector (*3*,*4*). Although studies in eastern Sudan have found domestic animals infected with the parasite (*5*,*6*), whether these animals play a role as disease reservoirs has not yet been proven; thus, transmission is still thought to be anthroponotic.

The disease was first reported from Southern Sudan in 1904, and the first epidemic was documented in 1940 with a death rate of 80% (*7*). Beginning in 1984, an epidemic (unrecognized until 1988) devastated the western part of Upper Nile state, ultimately causing ≈100,000 deaths in a population of 280,000 over a 10-year period (*3*).

**Figure 2:**
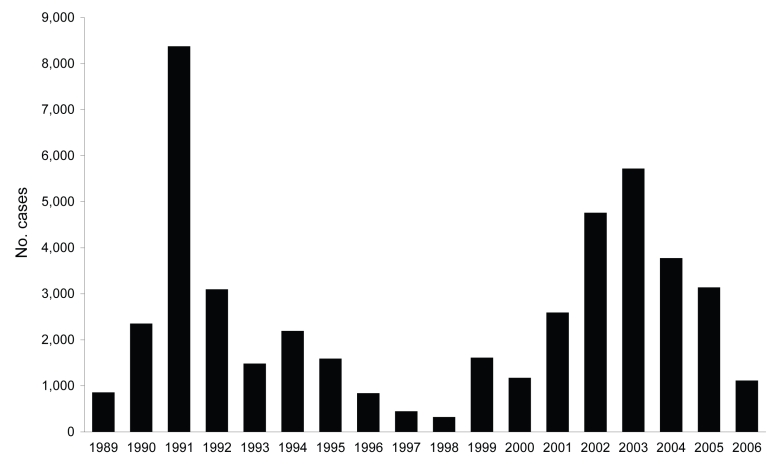
Total annual number of kala-azar cases in Southern Sudan reported to the World Health Organization, 1989–2006.

Passive case-detection data on kala-azar in Southern Sudan, collected by the World Health Organization (WHO) since 1989, indicate a cyclical pattern of kala-azar with considerable variation in the caseload from year to year (Figure 2). The dynamics presented in Figure 2 also suggest that Southern Sudan is currently between epidemics and provide a warning that cases may rise dramatically in coming years. In 2006, a total of 1,117 cases were reported, 65.4% of which were primary cases; the remainder were either relapses or cases of post–kala-azar dermal leishmaniasis. From January through June 2007, a total of 492 cases were reported, of which 88.2% were primary cases. The 5 locations accounting for 74.2% of the primary cases in 2007 were Malakal (n = 83), Ulang (n = 72), Nasir (n = 63), and Kiechkuon (n = 25) in Upper Nile state and Lankien (n = 79) in northern Jonglei state. Since 2002, the case-fatality rate recorded at healthcare facilities has been 4%–6%.

These data likely underestimate the actual number of cases because healthcare providers do not always provide complete reports and many kala-azar patients never visit healthcare facilities. Epidemiologic modeling of data from Upper Nile state estimated that those who visited healthcare facilities from October 1998 through May 2002 represented only 55% of cases and that 91% of kala-azar deaths were undetected (*8*). Health coverage is so minimal that some patients must walk for several days to access even the most basic healthcare services.

Despite the availability of different rapid diagnostic tests, most facilities used clinical diagnosis alone until recently. Only a few had the supplies and equipment to confirm suspected cases through microscopic examination of lymph node aspirate, which has a sensitivity of only 53%–65% (*1*). Nongovernment healthcare providers and Government of Southern Sudan–administered healthcare facilities thus had to confirm suspected kala-azar by direct agglutination test before they could receive a supply of the first-line treatment—sodium stibogluconate—from WHO through Pharmaciens sans Frontières. The use of the direct agglutination tests was required because of concerns about the sensitivity and specificity of the rK39 dipsticks in East Africa; a recent study suggests that these concerns were well founded (*9*). Also, many facilities that had received dipsticks were not using them. Until 2004, many healthcare facilities did not have the equipment or skills to conduct direct agglutination tests, and blood samples had to be sent to Kenya for analysis, which often led to treatment delays as long as 18 days. Now some healthcare facilities can analyze these samples internally and start treatment within 24 hours.

Confirmed cases are treated with sodium stibogluconate at a dose of 20 mg/kg/day for 30 days. Recently, because of an increasing number of patients in Upper Nile state who were nonresponsive to sodium stibogluconate, Médicins sans Frontières tested a combination of sodium stibogluconate and paromomycin, which would reduce treatment duration (from 30 to 17 days) and cost. Patient survival and initial cure rates were better than those for patients who received sodium stibogluconate monotherapy (*10*). However, completion of multicountry phase III trials being conducted by the Drugs for Neglected Diseases Initiative (www.dndi.org) is eagerly awaited before the combination can be considered as an alternative. Amphotericin B, a second-line drug for treatment of kala-azar, is not yet available in Southern Sudan’s facilities except in those run by Médicins sans Frontières.

Much remains unknown about the epidemiology of kala-azar in Southern Sudan (*11*). In the absence of detailed information on risk factors (cultural, demographic, epidemiologic, clinical, and geographic), use of long-lasting insecticide-treated nets seems a suitable method of prevention. Results from studies in Northern Sudan showed that insecticide-treated nets provided 27% protection from kala-azar (*12*). Whether similar protection can be achieved in Southern Sudan’s disease-endemic areas requires confirmation because effectiveness is dependent on human and vector behavior (*13*).

The return of stability to Southern Sudan has opened up new challenges and opportunities for kala-azar control. Large-scale population movement of susceptible or infected populations into kala-azar–endemic or –nonendemic areas respectively, poses a major epidemic risk. The healthcare systems are weak and rely on support from faith-based and nongovernment organizations, which need to be coordinated to ensure consistency in diagnosis, treatment, and prevention. As health infrastructure and human resources are being built up, kala-azar will need to be addressed as an integral part of multifunctional healthcare delivery by government staff, but this requires training and the provision of essential supplies.

Kala-azar falls under the mandate of the Director General of Preventive Medicine within the Ministry of Health–Government of Southern Sudan. The Ministry of Health, with support from WHO and in conjunction with nongovernment organizations working on kala-azar, has embarked on a number of activities to strengthen case-management. Laboratory technicians in most referral facilities have now been trained on the direct agglutination test; case-management guidelines have been updated; the essential drugs list is being reviewed and expanded to include alternatives for second-line treatment; and rK39 dipsticks are being distributed to peripheral health facilities to complement clinical diagnosis. With the revision of diagnosis and treatment guidelines, facilities are now able to obtain sodium stibogluconate by providing Pharmaciens sans Frontières with a positive rapid diagnostic test result, but they are encouraged to also take a blood sample for direct agglutination testing, as this is still considered more reliable (*8*). Meanwhile, the UK-based Malaria Consortium is providing long-lasting insecticide-treated nets to areas in Jonglei and Eastern Equatoria, where malaria and kala-azar are co-endemic.

## Conclusions

A strong presence of international donors and the Southern Sudan government’s desire to quickly reconstruct the healthcare sector provide ample opportunity to reduce the incidence of kala-azar. However, this goal can be achieved only with the necessary resources.
